# Construction of BHV-1 UL41 Defective Virus Using the CRISPR/Cas9 System and Analysis of Viral Replication Properties

**DOI:** 10.3389/fcimb.2022.942987

**Published:** 2022-07-08

**Authors:** Haiyue Dai, Jianan Wu, Hongshu Yang, Yongli Guo, Haoqing Di, Mingchun Gao, Junwei Wang

**Affiliations:** Heilongjiang Provincial Key Laboratory of Zoonosis, Department of Preventive Veterinary Medicine, College of Veterinary Medicine, Northeast Agricultural University, Harbin, China

**Keywords:** BHV-1, UL41, CRISPR/Cas9, defective virus, replications

## Abstract

Bovine herpesvirus type 1 (BHV-1) is a neurotropic herpesvirus that causes infectious rhinotracheitis and vulvovaginitis in cattle. The virion host shutoff protein encoded by the BHV-1 *UL41* gene is highly conserved in the Alphaherpesvirinae subfamily. This protein can degrade viral and host messenger RNA (mRNA) to interrupt host defense and facilitate the rapid proliferation of BHV-1. However, studies on the BHV-1 *UL41* gene are limited, and BHV-1 defective virus construction using the CRISPR/Cas9 system is somewhat challenging. In this study, we rapidly constructed a BHV-1 UL41-deficient strain using the CRISPR/Cas9 system in BL primary bovine-derived cells. BHV-1 UL41-defective mutants were screened by Western blot analysis using specific polyclonal antibodies as the primary antibodies. During the isolation and purification of the defective strain, a mixed virus pool edited by an efficient single-guide RNA (sgRNA) showed a plaque number reduction. Viral growth property assessment showed that BHV-1 UL41 was dispensable for replication, but the UL41-defective strain exhibited early and slowed viral replication. Furthermore, the BHV-1 UL41-deficient strain exhibited enhanced sensitivity to temperature and acidic environments. The BHV-1 UL41-deficient strain regulated viral and host mRNA levels to affect viral replication.

## Introduction

Bovine herpesvirus type 1 (BHV-1) is a pivotal member of the order Herpesvirales, family Herpesviridae, subfamily Alphaherpesvirinae, and genus *Varicellovirus* (Ackermann and Engels, 2006). BHV-1 has a relatively short reproductive cycle, spreads rapidly in culture, efficiently destroys infected cells, and can quickly establish latent infections not only in ganglia but also in other tissues (Valyi-Nagy et al., 2007). Upon entry into the bovine host, BHV-1 remains latent but infects susceptible animals when it is reactivated (Weidner-Glunde et al., 2020). One characteristic of herpesvirus-infected cells is the rapid shutoff of host macromolecular metabolism at the early stage of infection (Smith et al., 2000). Host protein synthesis also declines very rapidly after infection, and this decline is accompanied by cessation of glycosylation of host proteins. Although some viral proteins are produced late in infection, they expressed functions early in viral replication (Wentink et al., 1993). The host shutoff protein synthesis occur immediately after infection, and structural proteins release viral DNA from the capsid during nuclear processing.

The *UL41* gene of BHV-1 appears to be non-essential for the multiplication in cell culture under non-selective conditions. This gene encodes the virion host shutoff (VHS) protein, a late tegument protein expressed in BHV-1 infections. The VHS protein has a function similar to that of mRNA-specific ribonuclease (RNase) and can induce host shutoff early in infection (Taddeo and Roizman, 2006). However, VHS does not effectively discriminate between proteins synthesized by viruses and those synthesized by the host in its shutoff process. VHS degrades not only mRNAs from host cells to evade host abrogation but also mRNAs from viruses to facilitate the sequential expression of various classes of viral genes (He et al., 2020). Its unrestrained RNase activity is lethal but can be neutralized *via* formation of a trimeric complex with the tegument proteins VP22 and VP16 at the late stage of infection (Elliott et al., 2018). VP13/14, another viral protein, also plays a vital role in protecting against the degradation of viral transcripts (Shu et al., 2013a; Shu et al., 2013b). The VHS homology protein in varicella-zoster virus, a late protein encoded by open reading frame (ORF) 17, induces delayed shutoff of cellular RNA translation (Desloges et al., 2005). The VHS homology protein of equid herpesvirus type 1 encoded by ORF19 cannot degrade mRNAs or induce the shutoff of cellular and viral protein synthesis (Stokol and Soboll, 2019). The VHS homology protein in Pseudorabies virus (PRV) induces host mRNA degradation and cleaves the internal ribosomal entry site (IRES) sequence containing RNA (Lin et al., 2010; Liu et al., 2016). The VHS homologous protein of DPV influences pol II mRNA degradation, protein synthesis shutoff, and viral replication and spread (He et al., 2021). The VHS homologous protein of BHV-1 degrades the 5′ cap and 3′ untranslated region (UTR) adenylate-uridylate (AU)-rich element (ARE) areas of STAT1 in the antiviral pathway (Ma et al., 2019).

Clustered regularly interspaced short palindromic repeats (CRISPR)/CRISPR-associated protein 9 (Cas9), a revolutionary gene engineering technology, has recently been used to successfully edit various viruses (Borca et al., 2018; Das et al., 2019; Neuhausser et al., 2020; Tang et al., 2021; Van Cleemput et al., 2021). For example, HIV-1 (Das et al., 2019), herpes simplex virus type 1 (Velusamy et al., 2020), Marek’s disease virus (Luo et al., 2020), African swine fever virus (Borca et al., 2018), and PRV (Van Cleemput et al., 2021) have been extensively edited with the CRISPR/Cas9 system. The method is straightforward; the only requirement is an effective single-guide RNA (sgRNA) that targets the given gene with a protospacer-adjacent motif (PAM) sequence. The greatest disadvantages of this technique for BHV-1 editing are the plasmid’s low transfection efficiency, the unsustainable cell platform (Yang et al., 2019), and the high GC content in the BHV-1 genome. Even though the DNA viral genome can easily be edited, few BHV-1 deficiency mutants have been successfully constructed (Lobanov et al., 2010). To date, the CRISPR/Cas9 system has not been used to efficiently or rapidly construct a defective BHV-1 strain. In this study, we attempted to construct a BHV-1 UL41 strain using the CRISPR/Cas9 system in bovine lung (BL) cells and further explore the replication properties of the deficient strain.

## Materials and methods

### Cells, Viruses, and Animals

Madin–Darby bovine kidney (MDBK) cells and primary embryonic BL cells were stored at our laboratory and cultured in Dulbecco’s modified Eagle’s medium (DMEM) with 10% heat-inactivated fetal bovine serum (FBS, Gibco, Life Technologies) and antibiotics (streptomycin, 100 μg/ml; penicillin, 100 IU/ml; and amphotericin B, 0.25 μg/ml). All culture media were maintained in an incubator with 5% CO_2_ at 37°C. The BHV-1 strain was obtained from the China Institute of Veterinary Drug Control (Beijing, China). Three New Zealand White rabbits (8 weeks old) were purchased from the Laboratory Animal Center of Harbin Veterinary Research Institute. The animal treatment protocols followed the Chinese Regulations of Laboratory Animals and the Guidelines for the Care of Laboratory Animals. The experiments on rabbits were approved by the Laboratory Animal Ethical Committee of Northeast Agricultural University.

### Preparation and Detection of Rabbit Polyclonal Antibodies

An epitope of BHV-1 UL41 was designed and ligated into the pET30a(+) vector. The cloning and expression primers are shown in **Table 1**. Recombinant protein expression was induced with 1 μmol/L isopropyl β-d-1-thiogalactopyranoside (IPTG). The purified recombinant BHV-1 UL41 protein was collected from the gel and used as the immunogen to immunize rabbits. Specifically, after three freeze–thaw cycles, the gelled UL41 protein was injected subcutaneously. Two and four weeks after the first immunization, two more rounds of enhanced immunization were performed. Two weeks after the last immunization, the postimmunization serum containing polyclonal antibodies were collected. The rabbit anti-BHV-1 UL24 and anti-BHV-1 VP24 polyclonal antibodies were prepared in the same manner as the rabbit anti-BHV-1 UL41 polyclonal antibodies.

**Table 1:** Cloning and expression primers used in this study.

Primers	Sequence (5’–3’)
UL41-F	CGGCGCTTTCGCTCGCCTCTTA
UL41-R	CGCCTCCTGGGACCGATTT
UL24-F	CAGGTAGATACGCACGACGCGGAGA
UL24-R	TACAAAGACGCGGTCCGCGACTGCG
VP24-F	GCCAACCTGACGTTCCTCTGCG
VP24-R	CACCGTGTTATTTGCGGCTGTTT
30a-UL41-F	CGC*GGATCC*CGCGGCATCCACGGG
30a-UL41-R	CCG*CTCGAG*TTAGAGCCGAGGGTCGGG
30a-UL24-F	GC*GGATTC*CTGCAAAGCCGGCGGCCCGATT
30a-UL24-R	CG*CTCGAG*ATTGCCGCCCGACGCGTCTTTA
30a-VP24-F	GG*GGTACC*GCCCCCTCGCTCACGC
30a-VP24-R	CCC*AAGCTT*ATTAGCGTGCGACGGTGGCGG

The sensitivity of the antibodies was detected with ELISA. Antigen (50 ng/well Ni^+^ chromatographic affinity-purified recombinant protein) was coated onto the bottom of a 96-well ELISA plate, and 5% skimmed milk was added. The plate was incubated at 37°C for 1 h. The plate was rinsed three times with 1× PBST, and diluted polyclonal antibodies were added to the wells. The samples were incubated at 37°C for another hour and then rinsed three more times. A goat anti-rabbit horseradish peroxidase (HRP)-conjugated secondary antibody (ZB-2301, ZSGB-BIO, Beijing, China) was added, and the plate was incubated for 45 min at 37°C in an incubator. Then, the plate was rinsed, and 3,3′5,5′-tetramethylbenzidine (TMB) solution was used to visualize the HRP. Finally, 2 M H_2_SO_4_ was used as the stop solution to end the reaction. The results were read at 450 nm with a microplate reader.

The specificity of the polyclonal antibodies was detected by Western blotting. Recombinant proteins were separated by sodium dodecyl sulfate–polyacrylamide gel electrophoresis (SDS-PAGE) and transferred to nitrocellulose (NC) filter membranes (Pall Corporation, USA) with the prepared antibodies as the primary antibodies. A goat anti-rabbit HRP-conjugated antibody was used as the secondary antibody. A GeneGnome XRQ chemiluminescence imaging system (Syngene, UK) was used for image generation. A mouse anti-Flag antibody (M20008XS), mouse anti-Actin monoclonal antibody (M20003XS), and mouse anti-β-tubulin antibody (M20005XS) were purchased from Abmart (Shanghai, China).

### Generation of CRISPR/Cas9 sgRNA plasmids

Three sgRNAs targeting the ORF of the UL41 gene were designed using the online CRISPR Design Tool (CRISPR gRNA Design tool - ATUM). The oligo sequences of the three sgRNAs are listed in **Table 2**. Recombinant plasmids were constructed. Briefly, the forward and reverse primers of the oligos sgRNA_UL41_1–3 were annealed, and the oligos were ligated into the *Bbs*I-digested pX330-U6-Chimeric_BB-CBh-hSpCas9 vector. The recombinant plasmids were transformed into DH5α competent cells to amplify the constructs. All constructs were verified by BGI sequencing. Trans2K (BM101) and 15K (BM161) were purchased from TransGen Biotech (Beijing, China).

**Table 2:** The targeting sgRNA oligo sequences.

Oligos	Sequence (5’–3’)
sgRNA-1-F	CACCGATGTGCCAGCTTGGGCGCGT
sgRNA-1-R	AAACACGCGCCCAAGCTGGCACATC
sgRNA-2-F	CACCGCTTGGGCGCGTTGGCCCGCG
sgRNA-2-R	AAACCGCGGGCCAACGCGCCCAAGC
sgRNA-3-F	CACCGTACGCGTAACGCAGTAGCTT
sgRNA-3-R	AAACAAGCTACTGCGTTACGCGTAC

### Gene Inactivation By CRISPR/Cas9

BL cells were seeded in six-well plates and transiently transfected 12 h later with CRISPR/Cas9 recombinant plasmids containing sgRNA using a 2× Max transfection reagent (purchased from Harbin Medical University, China). Before transfection, the 2× Max transfection reagent needed to be preheated at 56°C for 5–10 min. The transfection procedure was as follows: dilute 4 μg of DNA to 200 μl with serum-free DMEM; add 8 μl of 2 mg/ml of the Max transfection solution and vortex immediately; let it stand at room temperature for 10 min; and add the mixture to BL cells with 800 μl medium (with serum). Twelve hours post-transfection, the cells were inoculated with BHV-1at an multiplicity of infection (MOI) of 0.01. Forty-eight hours later, the supernatants were purified by plaque formation assay. Several plaques were randomly selected and placed in 48-well cell culture plates. After virus amplification, the cell lysates were analyzed by Western blotting. The supernatants were saved as the primary virus. The viral DNA of the UL41-defective strain was extracted using an AxyPrep Body Fluid Viral DNA/RNA Miniprep Kit (Axygen, USA) according to the manufacturer’s instructions. Inactivation-specific genes were confirmed by DNA sequencing.

### Effective sgRNA Screening

The resulting virus pools were screened by plaque formation assay and Western blotting. For the plaque formation assay, MDBK cells were seeded the six-well plates and starved for 1 h with serum-free DMEM. Then, serial dilutions (10^−2^- to 10^−6^-fold) of BHV-1 were injected with the cells for 2 h. The cells were rinsed three times with PBS, and the empty plates were overlaid with 0.7%–0.8% low-melting-point agarose (Amrosco, USA) mixed with DMEM and 2% FBS. The plates were further incubated at 37°C for 3 days. Then, the plates were stained with 0.5% neutral red and covered with a tinfoil in a 37°C incubator for 30 min. The plaque numbers were calculated in triplicate. Plaques were picked and inoculated into new cell culture plates to expand the viruses. Western blotting was conducted. Briefly, cells were collected, rinsed three times with PBS, and then lysed in RIPA lysis buffer (Beyotime Biotechnology, China) containing a protease inhibitor cocktail (Beyotime). The proteins contained in the cell lysates were separated by SDS-PAGE, and all proteins were transferred to the NC filter membranes. Prepared polyclonal antibodies were used as the primary antibodies to analyze viral protein expression. The defective virus (based on the protein expression levels) that was edited by the most effective sgRNA was selected in this study.

### *In vitro* Growth Property Assessment

Wild-type (WT) BHV-1 or BHV-1 UL41^-^ was used to infect MDBK cells at an MOI of 0.01. The cells were harvested 6, 12, 24, 36, 48, 60, and 72 h after infection. Serially diluted viruses (10^−1^- to 10^−8^-fold) were used to infect the cells at 37°C for 2 h. A PBS rinse was used to remove the excess viral inoculum. Then, 100 μl of 2% FBS DMEM was added to each well, and the cells were further cultured for 3 days. The cytopathic effects were recorded every day, and the tissue culture infective dose (TCID_50_) value was calculated with the Reed–Muench method. The viral titers were recorded as the TCID_50_/ml values at various time points.

### Analysis of Viral Environmental Stability

The relative resistance levels of the virus strains to pH and temperature were compared. For acid–base stability analysis, the WT and BHV-1 UL41^−^ viruses were placed in a pH of 5.0 or 9.0 and then recovered to the normal pH of 7.8. For thermal stability analysis, the virus fluids were incubated at 42°C or 65°C for 30 min and then stored at 4°C for 24 h. The environmental stability was recorded as the viral titers, which were calculated with the Reed–Muench method.

### Quantitative Polymerase Chain Reaction Analysis (qPCR)

Total RNA was extracted with TRIzol, and first-strand cDNA was synthesized with NovoScript Plus All-in-one 1st Strand cDNA Synthesis SuperMix (gDNA Purge) according to the manufacturer’s instructions. Quantitative PCR (qPCR) was performed on a Real-time PCR Detection System (ABI 7500 qPCR thermal cycler, USA) with a NovoStart SYBR qPCR SuperMix Plus Kit (Novoprotein Scientific). The viral and host mRNA detection primers are shown in **Table 3**. The 20 μl reaction system contained 10 μl of 2× SYBR SuperMix, 6.4 μl of qPCR primers (2.5 μM), 0.4 μl of ROX Reference Dye II, 1 μl of cDNA template, and 2.2 μl of ddH_2_O. The reactions were carried out under the following thermal cycling conditions: 95°C for 30 s and 40 cycles of 95°C for 5 s and 60°C for 34 s. A dissociation curve analysis step was also performed. The 18S rRNA was amplified with specific primers to normalize amplification values of the triplicate samples. The comparative 2^−ΔCT^ and 2^−ΔΔCT^ methods were used to calculate the relative expression levels.

**Table 3:** Primers used in qPCR detection.

Oligos	Sequence (5’–3’)
q-UL34-S	GTGCGTCTTCCAGTTCAA
q-UL34-A	GCCATTAGCCGCAAGATG
q-UL54-S	CTGCGAGACCTGGTGCTG
q-UL54-A	TTCTTGGTGGCGATGAACTTG
q-UL47-S	CGCCGACGACTACGATAG
q-UL47-A	TGCGTCTGTGTCCATAGC
q-ISG15-S	GCAGCCAACCAGTGTCTG
q-ISG15-A	CCTAGCATCTTCACCGTCAG
q-Mx1-S	TCAACCTCCACCGAACTG
q-Mx1-A	TCTTCTTCTGCCTCCTTCTC
q-Viperin-S	TACACCCACGTCCAAGATGG
q-Viperin-A	TACACCCACGTCCAAGATGG
q-OAS-S	TTCGGTCATCTTGCTCTCAG
q-OAS-A	GTCTATCTCAACAGTCACAATCC
18S rRNA-S	TGTGATGCCCTTAGATGTCC
18S rRNA-A	TTATGACCCGCACTTACTGG

### Statistical Analysis

Graphs were created and relevant statistical tests were performed with GraphPad Prism version 9.0.0 (121) (GraphPad Software, CA, USA) in this work. Statistical analysis was performed by one- or two-way ANOVA methods. Values of *p < 0.05 were considered to indicate statistical significance.

## Results

### Generation of CRISPR/Cas9 sgRNA Plasmids and Construction of BHV-1 UL41^-^

**Figure 1:**
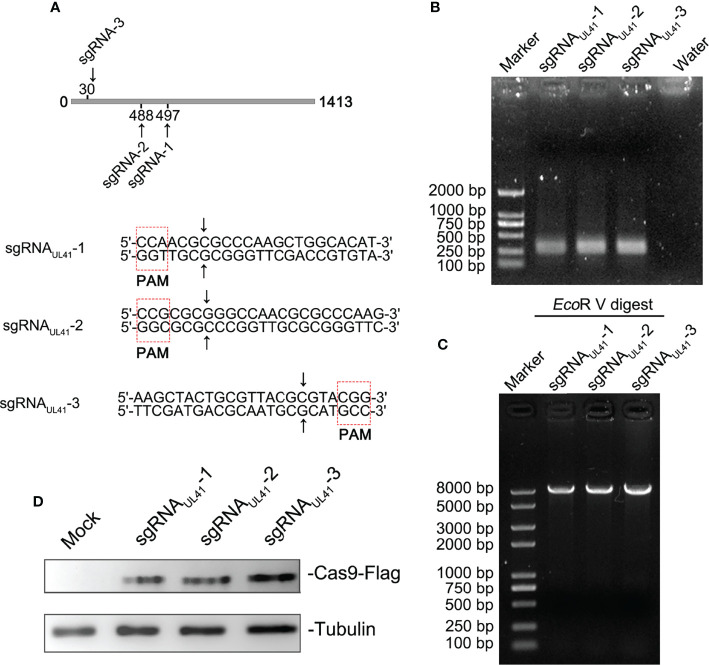
Generation of CRISPR/Cas9 sgRNA plasmids and construction of BHV-1 UL41^−^. **(A)** CRISPR/Cas9 sgRNA-targeted sequences and PAM array. Three sgRNA targeting positions are marked in the UL41 sequence diagram. The CRISPR/Cas9-predicted cleavage sites are indicated by arrows. The red dotted frame areas are the PAM arrays. **(B)** PCR amplification of recombinant plasmids sgRNA_UL41_1–3. The lanes are marked with different recombinant plasmids. Water was used for the negative control lane, and the DNA marker was a 2-kb molecular ladder. The molecular weight of the specific amplified fragment of the positive recombinant plasmids was 300 bp. **(C)** Restriction endonuclease digestion identification of recombinant plasmids. Digestion of the pX330 vector was performed with the *Eco*RV restriction enzyme, and the DNA marker was a 8-kb molecular ladder. **(D)** Analysis of the transfected CRISPR/Cas9 recombinant plasmids. Mock indicates BL cells treated with the transfection agent. Cas9 protein expression was detected with a mouse anti-Flag antibody. The three sgRNA recombinant plasmids are marked above the lane. Tubulin was the internal reference protein.

To investigate the importance of the *UL41* gene for the lytic cycle of BHV-1, we constructed a BHV-1 UL41 defective strain. Using online software, we designed three specific sgRNAs to target the UL41 ORF. The targeting sequences and PAM are shown in **Figure 1A**. The designed three sgRNAs targeted the first 500 bp N-terminal sequence of the *UL41* gene. The sgRNA_UL41_1–3 oligos were inserted into the pX330 vector and the U6-Forward primer was used with specific reverse primer for PCR amplification. The positive recombinant plasmids were identified by PCR (**Figure 1B**). The size of the positive amplification product was approximately 270 bp. To further confirm the insertion of the fragment into the pX330 vector, the restriction endonuclease *Eco*RV was used to digest the constructs, and the recombinant plasmids pX330-sgRNA_UL41_1–3 were identified (**Figure 1C**). The overall size of the pX330 vector and insert was 8,509 bp, and the digestion identification results were as expected. We next tested whether the CRISPR/Cas9 recombinants effectively disrupted the BHV-1 genome by transfecting BL cells. The cells were infected with BHV-1 (MOI=0.01) 12 h post-transfection. BL cells were collected after 48 h of infection, and the cell lysates were analyzed by Western blotting (**Figure 1D**). The Cas9-Flag protein indicated the recombinant plasmids pX330-sgRNA_UL41_1–3 were transfected into the BL cells, and the efficiency of transfection in BL was ideal.

### Detection Sensitivity and Specificity of Rabbit Anti-BHV-1 UL41 Antibodies

**Figure 2:**
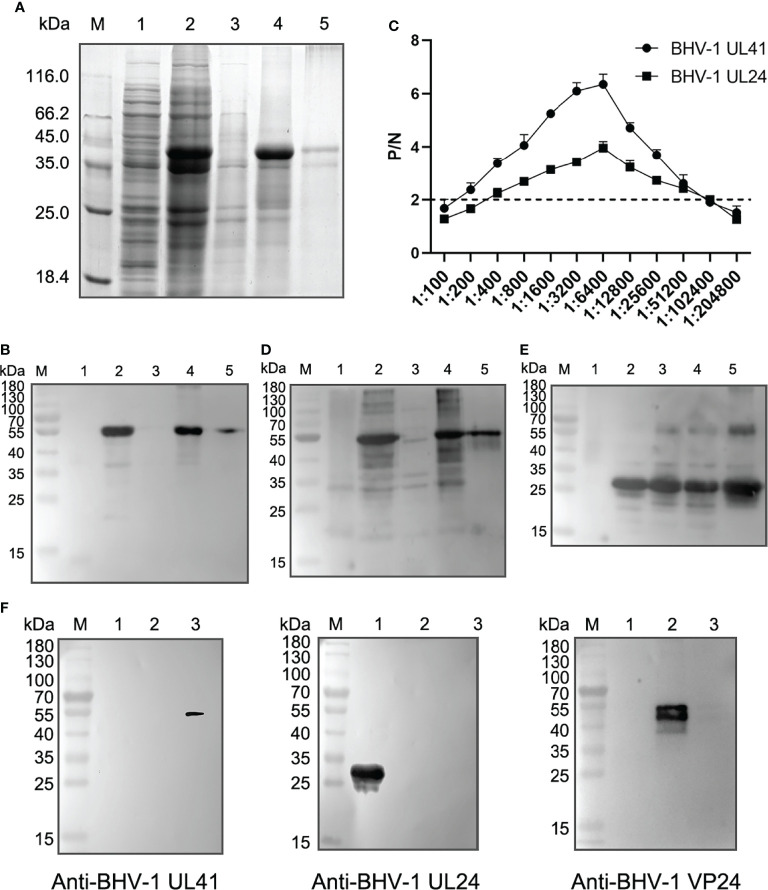
Detection sensitivity and specificity of rabbit anti-BHV-1 UL41 antibodies. **(A)** Expression analysis of rHis-BHV-1 UL41. Lane M: protein molecular ladder; lane 1: negative control of the pET30a(+) vector; lane 2: rHis-BHV-1 UL41 after induction; lane 3: supernatants of rHis-BHV-1 UL41; lane 4: sedimentations of rHis-BHV-1 UL41; lane 5: purified rHis-BHV-1 UL41. **(B)** Western blot analysis of rHis-BHV-1 UL41 expression. Notes of the lanes are the same as panel **(A)**. A mouse anti-His monoclonal antibody was used as the primary antibody. **(C)** ELISA of rabbit anti-rHis-BHV-1 UL41 and rabbit anti-rHis-BHV-1 UL24 polyclonal antibodies. P/N indicates the positive value/negative value ratio. The horizontal axis indicates the serum dilution. The dotted line indicates a P/N value of 2, which was the criterion value. **(D)** Specificity analysis of a rabbit anti-rHis-BHV-1 UL41 polyclonal antibody. Notes of the lanes are the same as panel **(A)**.The prepared rabbit anti-BHV-1 UL41 polyclonal antibody was used as the primary antibody. **(E)** Specificity analysis of a rabbit anti-rHis-BHV-1 UL24 polyclonal antibody. Lane M: protein molecular ladder; lane 1: negative control of the pET30a(+) vector; lane 2: rHis-BHV-1 UL24 after induction; lane 3: supernatants of rHis-BHV-1 UL24; lane 4: sedimentations of rHis-BHV-1 UL24; lane 5: purified rHis-BHV-1 UL24. The prepared rabbit anti-BHV-1 UL24 polyclonal antibody was used as the primary antibody. **(F)** Specificity analysis of the rabbit anti-rHis-BHV-1 polyclonal antibodies. Lane M: protein molecular ladder; lane 1: purified rHis-BHV-1 UL24; lane 2: purified rHis-BHV-1 VP24; lane 3: purified rHis-BHV-1 UL41. The primary antibody on the left is the rabbit anti-rHis-BHV-1 UL41 polyclonal antibody; that in the middle is the rabbit anti-rHis-BHV-1 UL24 polyclonal antibody; and that on the right is the rabbit anti-rHis-BHV-1 VP24 polyclonal antibody.

To screen the recombinant defective mutants by Western blotting, we prepared sensitive and specific antibodies against BHV-1 viral proteins. Specifically, we prepared a rabbit anti-BHV-1 UL41 polyclonal antibody to further detect UL41 expression. Rabbit anti-BHV-1 UL24 and anti-BHV-1 VP24 polyclonal antibodies were prepared as BHV-1 infection-positive controls. The BHV-1 VP24 protein is encoded by the BHV-1 UL26 gene. The ectopic expression of UL41 protein was mainly expressed as an inclusion body with a molecular mass of 52 kDa in the Coomassie blue protein gel (**Figure 2A**), and the purified UL41 protein was prepared as the immunogen to immunize rabbits. The ectopic expression of UL41 protein was expressed with a His-tagged protein in the N-terminus of the insert sequence. **Figure 2B** shows that the ectopic expression of protein was detected with the mouse anti-His monoclonal antibody by Western blotting. Ectopically expressed UL24 showed soluble and inclusion body expression, and it was expressed with a molecular mass of 32 kDa. When the immunization schedule was finished, serum was collected and used as the polyclonal antibody. The sensitivity of the polyclonal antibody was detected using ELISA, and the P/N value was used to evaluate the serum titer. In our results, P/N=2 was used as the sensitivity criterion, and 1:102,400 was the serum-sensitive dilution of the rabbit anti-BHV-1 UL41 and anti-BHV-1 UL24 polyclonal antibody (**Figure 2C**). The specificity of the polyclonal antibody was detected by Western blotting. As shown in **Figures 2D, E**, the prepared antibodies reacted more strongly with the ectopic expression of proteins than with the vector protein, which indicated well specificity of the rabbit anti-BHV-1 UL41 and anti-BHV-1 UL24 polyclonal antibody. To identify the specificity of the rabbit anti-BHV-1 UL41 polyclonal antibody, the ectopic expression of UL24 and VP24 were used as the irrelevant antigens for detection (**Figure 2F**). The rabbit anti-BHV-1 UL41 polyclonal antibody exhibited good sensitivity and specificity in detection.

### Effective sgRNA Screening and Identification of BHV-1 UL41^-^

**Figure 3:**
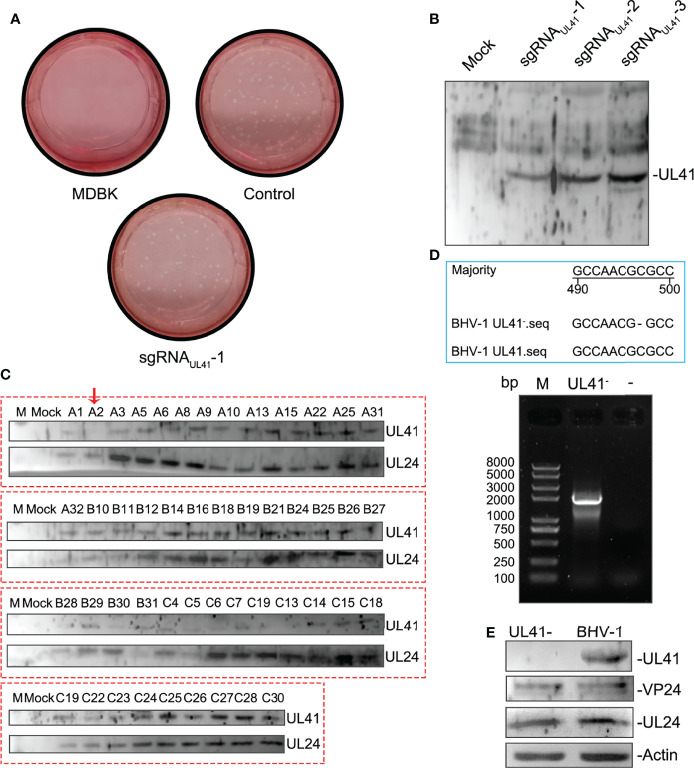
Effective sgRNA screening and identification of BHV-1 UL41^−^. **(A)** sgRNA_UL41_-1 inhibited BHV-1 plaque formation. BL cells were transfected with 4 μg of sgRNA_UL41_-1, sgRNA_UL41_-2, or sgRNA_UL41_-3. Twelve hours later, the cells were infected with BHV-1 (MOI=0.01), and the supernatants were subjected to a plaque formation assay after 48 h of infection. “Control” indicates the supernatants edited by the plasmids sgRNA_UL41_-2 and sgRNA_UL41_-3 for the plaque formation assay. The plaque numbers were calculated in three independent assays, ** for *p* < 0.01. **(B)** sgRNA_UL41_-1 inhibited BHV-1 UL41 expression. The cell lysates from panel **(A)** were subjected to Western blotting. UL41 expression was detected with the prepared polyclonal antibodies. “Mock” indicates blank BL cells. sgRNA_UL41_-1–3 indicates the cells infected with the sgRNA-Cas9-edited viruses. **(C)** BHV-1 UL41^−^ screening. Plaques from the sgRNA_UL41_-1 group were inoculated into 48-well plates. The cell lysates were collected after virus amplification and analyzed by Western blotting. A2 indicates the UL41-silenced expression virus and is marked with a red arrow. Letters A–C before the numbers indicate the plaques that were amplified from the three sgRNAs (A for sgRNA_UL41_-1, B for sgRNA_UL41_-2, and C for sgRNA_UL41_-3). BHV-1 UL24 and UL41 were detected with the prepared polyclonal antibodies. UL24 was used as the BHV-1 infection reference control. **(D)** Cloning and sequencing analysis of the defective viral UL41 gene. Lane M: Trans 2K Plus II DNA ladder. The blue-line-framed area represents the 490- to 500- nucleotide sequencing results of BHV-1 UL41^−^; “−” in the BHV-1 UL41^−^ sequence indicates an absent nucleotide. The “−” above the agarose gel chart indicates the water negative control. UL41^−^ indicates the UL41 gene amplification of the deficient virus. **(E)** Western blot analysis of the BHV-1 UL41^−^ strain. “UL41^−^” indicates the BHV-1 UL41^−^ strain, and BHV-1 indicates the parent strain. UL41, VP24, and UL24 were detected by the prepared polyclonal antibodies. Actin was used as the internal reference control; UL24 and VP24 were used as the BHV-1 infection reference controls.

The supernatants were serially diluted and used to infect MDBK cells to determine the effective sgRNA of BHV-1 UL41. A plaque formation assay was performed to assess the ability of the three groups of sgRNAs to target BHV-1 UL41. The numbers of viral plaques were significantly lower in the supernatants of the groups subjected to editing with sgRNA_UL41_-1 than in those of the other two groups (**Figure 3A**), indicating that the BHV-1 genome was effectively disrupted by sgRNA_UL41_-1. UL41 expression was relatively decreased under sgRNA_UL41_-1 editing after BHV-1 infection for 48 h (**Figure 3B**). Next, we tested whether the DNA breaks induced inactivating indels at the targeted cleavage site. Several plaques were picked and used to inoculate the new MDBK plates. The virus was amplified, and UL41 expression was analyzed. Our results revealed that the A2 strain presented significantly defective UL41 expression (**Figure 3C**). Thus, the CRISPR/Cas9 system successfully disrupted the BHV-1 genome and induced indels. We further selected a purified A2 isolate for PCR-based sequencing. The UL41 gene of the A2 strain was successfully cloned, and it contained a deletion of “C” at the 497th nucleotide according to sequencing by BGI (**Figure 3D**). Thus, a UL41 gene-inactivated BHV-1 strain was successfully constructed (hereafter named BHV-1 UL41^−^). The silencing of UL41 protein expression was confirmed by Western blot analysis compared to the expression in the WT strain. The correct expression of the UL24 and VP24 proteins in BHV-1 UL41^−^ (**Figure 3E**) indicated that sgRNA_UL41_-1 had good target specificity.

### Evaluation of Viral Replication and Environmental Stability

**Figure 4:**
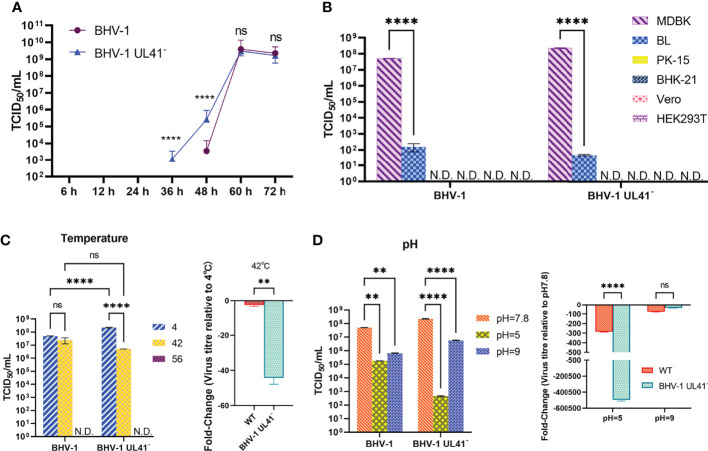
Evaluation of viral replication and environmental stability. **(A)** One-step growth curve of the BHV-1 UL41^−^ and WT viruses. MDBK cells were infected with viruses at an MOI of 0.01, and the viruses were collected after inoculation for 6, 12, 24, 36, 48, 60, and 72 h. The viral titers were recorded as TCID_50_/ml values. The blue triangle indicates the growth curve of BHV-1 UL41^−^, and the purple circle indicates the growth curve of the WT strain. **(B)** Cell tropism analysis of BHV-1 UL41^−^ and the WT strain. MDBK, BL, PK-15, BHK-21, Vero, and HEK293T cells were used to detect the cell tropism of the viruses. N.D. stands for not detected. **(C)** Temperature sensitivity analysis of BHV-1 UL41^−^ and the WT strain. The number 4 indicates that the viruses were collected and stored at 4°C for temperature stability detection. The number 42 indicates that the viruses were kept at 42°C for 30 min and cold at 4°C. The number 56 indicates that the viruses were kept at 56°C for 30 min and cold at 4°C. The data are plotted as the fold change over 4°C. N.D. stands for not detected. **(D)** pH sensitivity analysis of BHV-1 UL41^−^ and the parent strain. pH 7.8 indicates the pH of the initial viral solution; pH 5 indicated that the viruses recovered to pH 7.8 from pH 5; pH 9 indicates that the viruses recovered to pH 7.8 from pH 9; and the data are plotted as the fold change over pH 7.8. The viral titers were detected in three independent experiments and calculated as the mean ± SD. **** for *p* < 0.0001, ** for *p* < 0.01, and ns for no significance.

To determine whether UL41 inactivation affected BHV-1 replication, the replication properties of the virus were analyzed. MDBK cells were separately infected with BHV-1 UL41^−^ and the WT strain at an MOI of 0.01. As depicted in **Figure 4A**, the growth property of BHV-1 UL41^−^ resembled that of the WT strain. One-step growth curves indicated that the defective mutant replicated slower than the parent virus from 36 to 60 h and that it replicated earlier than the WT strain. The WT strain did not show replication at 36 h, as shown in **Figure 4A**. Next, we tested the cell tropism of BHV-1 in various research cell lines. **Figure 4B** shows that the virus was able to infect the two bovine-derived cell lines, while the MDBK cells presented enhanced adaptability to BHV-1 infection. The viral titer of BHV-1 replication in BL cells was lower (by almost 10^5^-fold) than that in MDBK cells. BHV-1 UL41^−^ showed the same cell tropism with the WT strain. To investigate the relative resistance of the defective and WT strains to temperature and pH variations, the viral titers were measured after the environment was changed. The replication of the virus slightly declined with increasing temperature (**Figure 4C**). However, the titer of the defective strain was 40-fold lower than that of the WT strain after the viruses recovered from 42°C exposure. The viruses were inactivated after recovery from 56°C. They replicated relatively stably in the low-temperature environment. BHV-1 UL41^−^ exhibited enhanced sensitivity to the pH 2.0 environment; the viral titer in this strain decreased by 10^5^-fold, while the titer in the WT strain decreased by only 200-fold after recovery from the pH 2.0 environment. Both BHV-1 UL41^−^ and the WT strain presented alkaline resistance characteristics after recovery from the pH 9.0 environment (**Figure 4D**). The viral titers of BHV-1 UL41^−^ and the WT strain were 100-fold lower than those under pH 7.8. In our research, BHV-1 UL41^−^ and the WT strain showed different acid–base stabilities in MDBK infections, which also indicated that the viruses could not stably stay in an overly acidic environment. We further tested the stability of BHV-1 UL41^−^ through 10 serial passages in MDBK cells. No reversion mutation was found by sequencing.

### The BHV-1 UL41-Deficient Strain Regulates Viral and Host mRNAs

**Figure 5:**
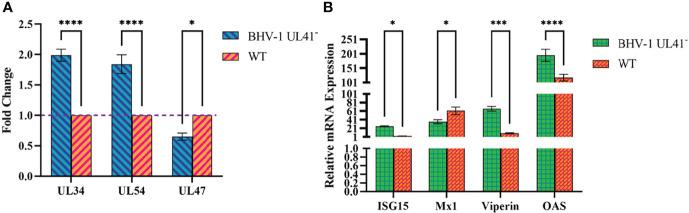
BHV-1 UL41^−^ regulates viral and host mRNAs. **(A)** BHV-1 UL41 regulates viral mRNA. The BHV-1 UL41^-^ and WT strains were used to infect MDBK cells at an MOI of 1, and total RNA was collected at 12 hpi. The mRNA levels of the immediate early gene UL34, the early gene UL54, and the late gene UL47 were analyzed using reverse transcription qPCR and normalized to those of 18S rRNA. The data are plotted as the fold change over the WT strain. **(B)** BHV-1 UL41 regulates host mRNA. The BHV-1 UL41^−^ and WT strains were used to infect MDBK at an MOI of 1, and total RNAs RNA was collected at 8 hpi. The relative gene expression levels were determined with the 2^−ΔΔCT^ method. The viral titers were detected in three independent experiments and calculated as the mean ± SD. **** for *p* < 0.0001, *** for *p* < 0.001, and * for *p* < 0.05.

To further investigate whether BHV-1 UL41 regulates viral and host mRNA levels to affect viral replication, MDBK cells were infected with BHV-1 UL41^−^ and the WT strain at an MOI of 1. Samples were collected and then assessed by reverse transcription qPCR. As shown in **Figure 5A**, compared with those in the WT strain, the mRNA levels of the immediate early (IE) gene UL34, the early (E) gene UL54, and the late (L) gene UL47 were changed in the BHV-1 UL41^−^-infected group. The levels of the viral IE and E genes were significantly increased, while those of the viral L gene were significantly decreased. Furthermore, we found that some host genes also exhibited significant changes in BHV-1 UL41^−^ infections (**Figure 5B**). Interferon-stimulated gene 15 (ISG15), Viperin, and oligoadenylate synthetase (OAS) mRNA levels were significantly increased, and Mx1 mRNA levels were significantly decreased. These results showed that stronger regulation of viral and host RNAs occurs following infection with BHV-1 UL41^−^ and indicate that BHV-1 UL41 broadly regulates viral and host mRNAs to affect viral replication.

## Discussion

Here, we used the BL primary cell line as the construction platform (Osorio and Bionaz, 2017), not only because it could be relatively well transfected with the targeting sgRNA CRISPR/Cas9 system plasmids but also because replication of BHV-1 could be achieved. We found a higher transfection efficiency of BL cells compared to MDBK cells (**Supplementary Figure S1**) and the cells cloud transfected as the normal primary cells with many transfection reagents. We had tried Lipofectamine 3000 and the Max transfection reagent in BL cells, and the transfection efficiency was similar. A specific sgRNA was used to rapidly edit the BHV-1 genome with the CRISPR/Cas9 system. Efficient sgRNAs were screened using polyclonal antibodies that specifically reacted with the expressed target protein expression by Western blotting. We simplified the construction and identification process, and the BHV-1 UL41^−^ strain was effectively screened using specific polyclonal antibodies. Although the CRISPR/Cas9 gene-editing system has some limitations for use in BHV-1 gene inactivation, previous research (Lobanov et al., 2010; Zhang et al., 2011; Sucharita et al., 2021) has revealed that a pivotal step is the discovery of highly transfectable cell lines that can be infected with the virus. The important challenges of CRISPR/Cas9 in genome editing were addressed by Zhang et al. (2014). One of the four issues was the delivery methods, and the efficiency of the delivery methods depends on the types of target cells and tissues. The other three challenges were off-target mutations, PAM dependence, and gRNA production. We selected the most efficient sgRNA for the given coding sequence. The construction method was rapid but had low efficacy. In our research, the UL41 gene was silently expressed in only one strain, so more convenient and highly mutagenic methods need to be studied.

The canonical mutations in a single nucleotide include insertions, substitutions, deletions, and subversions introduced into target genes. The strain under investigation, BHV-1 UL41^−^, appeared to have the single deletion of the nucleotide “C.” In this study, we attempted to identify the ORFs that target genes by using anti-UL41, anti-UL24, and anti-VP24 polyclonal antibodies to detect the expression of these genes. These antibodies were prepared in rabbits using the major antigenic domain of each ORF, and they had high sensitivity and specificity for the target proteins. We inactivated the UL41 ORF by introducing frameshift mutations; nucleotide-deletion-induced frameshift mutations are prone to silencing in WT sequences. In the future, to improve the viral reversion problems, large fragment deletions spanning the ORFs can be targeted in gene editing.

The ratio of gene disruption was determined according to the sgRNA efficacy. Although multiple CRISPR sequence online design tools can be utilized, many software programs depend fully on computational analysis, resulting in the design of numerous unclassified sgRNAs (Brazelton et al., 2015). In addition, the BHV-1 genome contains a high GC content, and only three advanced evaluated sgRNAs are available; those with higher scores are more effective. To obtain optimal results, the most effective sgRNA was selected for further study.

The biological characteristics of the BHV-1 UL41-defective strain in viral replication were examined. The BHV-1 UL41^−^ strain showed early viral replication, but it was slower than that of the parent strain. BHV-1 UL41 might shut off host proteins to promote viral replication at the early stage of infection and then interact with other viral proteins to interrupt host and virus shutoff functions. The interaction of UL41 with other proteins needs to be further explored. BHV-1 UL41^−^ was more sensitive to high temperature and acidic environments than the WT strain. Our results reveal that UL41 affects the physical and chemical characteristics of the virus, including the growth curve, temperature sensitivity, and acid–base tolerance. However, the main biological characteristics of the virus are not altered; that is, the virus can still be inactivated at 56°C and is relatively alkaline resistant.

The cell tropism experiment revealed that bovine-derived cells were susceptible to BHV-1 and that the MDBK cells were most suitable for use to evaluate viral replication and proliferation. Although the replication ability of BHV-1 was not as good in BL cells as it was in MDBK cells, BL cells still exhibited greater virus susceptibility than other animal-derived cells. MDBK cells are ideal for viral replication, but they have a challenging limitation for use in the construction of a gene-editing virus platform: the efficiency of MDBK cell transfection is low, approximately 1.7%–2.4% (Osorio and Bionaz, 2017), and the transfection efficiency of primary cells was higher than that of MDBK (**Supplementary Figure S1**). Therefore, BL cells were used as the platform for construction of the gene-editing virus, and MDBK cells were used as the evaluation model in the subsequent virus biological detection experiments.

BHV-1 UL34, UL54, and UL47 were the representative IE, E, and L genes, respectively (Robinson et al., 2008). ISG15, Mx1, Viperin, and OAS are important immune response molecules induced by interferon and play an important role in host resistance to pathogenic invasion. We selected these genes to investigate the effects of invasion by viruses. The reverse transcription qPCR results showed that BHV-1 UL41^−^ induced viral IE and E gene transcription at a relatively early stage and increased the host mRNA transcript levels. This result indicates that BHV-1 UL41 can inhibit viral IE and E gene transcription and decrease the host mRNA levels to facilitate viral replication.

In the present study, we rapidly knocked out the BHV-1 UL41 gene using the CRISPR/Cas9 gene-editing system. The viral replication levels of BHV-1 UL41^−^ and the WT strain were compared in multiple dimensions. The UL41-defective virus exhibited early but has slow viral replication and increased sensitivity to temperature and the acidity of the environment. The viral and host mRNA levels were regulated in the defective strain compared to the WT strain. Our strategy is pivotal for promoting BHV-1 control and prevention in China. Other viral genes of BHV-1 could also be considered for silencing expression by this method. In addition, efficient viral delivery systems such as lentivirus, adenovirus, or MultiBacMam could also be considered for delivery of CRISPR/Cas9 editing systems in BHV-1 genome editing. Based on the CRISPR/Cas9 gene-editing system, more viral gene functions of the BHV-1 genome could be explored.

## Data Availability Statement

The original contributions presented in the study are included in the article/supplementary material. Further inquiries can be directed to the corresponding authors.

## Ethics Statement

The animal study was reviewed and approved by the Laboratory Animal Ethical Committee of Northeast Agricultural University.

## Author Contributions

HYD wrote the first draft of the manuscript. MG and JWW contributed to the conception and design of the study. YG performed the statistical analysis. JNW organized the database. HQD and HY wrote sections of the manuscript. All authors contributed to manuscript revision, read, and approved the submitted version.

## Funding

We extend our sincere thanks to all the authors who have devoted themselves to this research. This work was supported by the National Natural Science Foundation of China (32002296) and China Agriculture Research System of MOF and MARA.

## Conflict of Interest

The authors declare that the research was conducted in the absence of any commercial or financial relationships that could be construed as a potential conflict of interest.

## Publisher’s Note

All claims expressed in this article are solely those of the authors and do not necessarily represent those of their affiliated organizations, or those of the publisher, the editors and the reviewers. Any product that may be evaluated in this article, or claim that may be made by its manufacturer, is not guaranteed or endorsed by the publisher.

## Supplementary Material

Supplementary Figure 1 The transfection efficiency of BL cells and MDBK cells. **(A)** Direct fluorescence analysis of the transfection efficiency of BL and MDBK. A plasmid of pcDNA3.1-EGFP transfected into BL and MDBK cells by 2× Max transfection reagent, **(B)** Western blot analysis of the transfection efficiency of BL and MDBK. Recombinant plasmids of pX330-sgRNA_UL41_1-3 were transfected into BL and MDBK cells by Lipofectamine 3000 reagent (Invitrogen, USA). Cas9-Flag indicated the expression of recombinant plasmids, and GAPDH was the internal reference protein.

## References

[B1] Ackermann M. Engels M. (2006). Pro and Contra IBR-Eradication. Vet. Microbiol. 113 (3-4), 293–302. doi: 10.1016/j.vetmic.2005.11.043 16337098

[B2] Borca M. V. Berggren K. A. Ramirez-Medina E. Vuono E. A. Gladue D. P. (2018). CRISPR/Cas Gene Editing of a Large DNA Virus: African Swine Fever Virus. Bio Protoc. 8 (16), e2978. doi: 10.21769/BioProtoc.2978 PMC8328649 34395778

[B3] Brazelton V. J. Zarecor S. Wright D. A. Wang Y. Liu J. Chen K.. (2015). a Quick Guide to CRISPR sgRNA Design Tools. GM Crops Food 6 (4), 266–276. doi: 10.1080/21645698.2015.1137690 26745836 PMC5033207

[B4] Das A. T. Binda C. S. Berkhout B. (2019). Elimination of Infectious HIV DNA by CRISPR-Cas9. Curr. Opin. Virol. 38, 81–88. doi: 10.1016/j.coviro.2019.07.001 31450074 PMC7050564

[B5] Desloges N. Rahaus M. Wolff M. H. (2005). The Varicella-Zoster Virus-Mediated Delayed Host Shutoff: Open Reading Frame 17 has No Major Function, Whereas Immediate-Early 63 Protein Represses Heterologous Gene Expression. Microbes Infect. 7 (15), 1519–1529. doi: 10.1016/j.micinf.2005.05.010 16039898

[B6] Elliott G. Pheasant K. Ebert-Keel K. Stylianou J. Franklyn A. Jones J. (2018). Multiple Posttranscriptional Strategies to Regulate the Herpes Simplex Virus 1 Vhs Endoribonuclease. J. Virol. 92 (17), e00818–18. doi: 10.1128/JVI.00818-18 PMC6096803 29925667

[B7] He T. Wang M. Cheng A. Yang Q. Jia R. Wu Y.. (2021). DPV UL41 Gene Encoding Protein Induces Host Shutoff Activity and Affects Viral Replication. Vet. Microbiol. 255, 108979. doi: 10.1016/j.vetmic.2021.108979 33721633

[B8] He T. Wang M. Cheng A. Yang Q. Wu Y. Jia R.. (2020). Host Shutoff Activity of VHS and SOX-Like Proteins: Role in Viral Survival and Immune Evasion. Virol. J. 17 (1), 68. doi: 10.1186/s12985-020-01336-8 32430029 PMC7235440

[B9] Lin H. W. Hsu W. L. Chang Y. Y. Jan M. S. Wong M. L. Chang T. J. (2010). Role of the UL41 Protein of Pseudorabies Virus in Host Shutoff, Pathogenesis and Induction of TNF-Alpha Expression. J. Vet. Med. Sci. 72 (9), 1179–1187. doi: 10.1292/jvms.10-0059 20448414

[B10] Liu Y. F. Tsai P. Y. Chulakasian S. Lin F. Y. Hsu W. L. (2016). The Pseudorabies Virus Vhs Protein Cleaves RNA Containing an IRES Sequence. FEBS J. 283 (5), 899–911. doi: 10.1111/febs.13642 26744129

[B11] Lobanov V. A. Maher-Sturgess S. L. Snider M. G. Lawman Z. Babiuk L. A. van Drunen L. D. H. S. (2010). A UL47 Gene Deletion Mutant of Bovine Herpesvirus Type 1 Exhibits Impaired Growth in Cell Culture and Lack of Virulence in Cattle. J. Virol. 84 (1), 445–458. doi: 10.1128/JVI.01544-09 19864376 PMC2798391

[B12] Luo J. Teng M. Zai X. Tang N. Zhang Y. Mandviwala A.. (2020). Efficient Mutagenesis of Marek’s Disease Virus-Encoded Micrornas Using a CRISPR/Cas9-Based Gene Editing System. Viruses 12 (4), 466. doi: 10.3390/v12040466 PMC7232411 32325942

[B13] Ma W. Wang H. He H. (2019). Bovine Herpesvirus 1 Tegument Protein UL41 Suppresses Antiviral Innate Immune Response *via* Directly Targeting STAT1. Vet. Microbiol. 239, 108494. doi: 10.1016/j.vetmic.2019.108494 31767068

[B14] Neuhausser W. M. Oh H. S. Eggan P. Angelova M. Kirchner R. Eggan K. C.. (2020). Screening Method for CRISPR/Cas9 Inhibition of a Human DNA Virus: Herpes Simplex Virus. Bio Protoc. 10 (17), e3748. doi: 10.21769/BioProtoc.3748 PMC7842287 33659408

[B15] Osorio J. S. Bionaz M. (2017). Plasmid Transfection in Bovine Cells: Optimization Using a Realtime Monitoring of Green Fluorescent Protein and Effect on Gene Reporter Assay. Gene 626, 200–208. doi: 10.1016/j.gene.2017.05.025 28501631

[B16] Robinson K. E. Meers J. Gravel J. L. McCarthy F. M. Mahony T. J. (2008). The Essential and non-Essential Genes of Bovine Herpesvirus 1. J. Gen. Virol. 89 (Pt 11), 2851–2863. doi: 10.1099/vir.0.2008/002501-0 18931083

[B17] Shu M. Taddeo B. Roizman B. (2013a). The Nuclear-Cytoplasmic Shuttling of Virion Host Shutoff RNase is Enabled by Pul47 and an Embedded Nuclear Export Signal and Defines the Sites of Degradation of AU-Rich and Stable Cellular Mrnas. J. Virol. 87 (24), 13569–13578. doi: 10.1128/JVI.02603-13 24109211 PMC3838220

[B18] Shu M. Taddeo B. Zhang W. Roizman B. (2013b). Selective Degradation of Mrnas by the HSV Host Shutoff RNase is Regulated by the UL47 Tegument Protein. Proc. Natl. Acad. Sci. U. S. A. 110 (18), E1669–E1675. doi: 10.1073/pnas.1305475110 23589852 PMC3645526

[B19] Smith T. J. Ackland-Berglund C. E. Leib D. A. (2000). Herpes Simplex Virus Virion Host Shutoff (Vhs) Activity Alters Periocular Disease in Mice. J. Virol. 74 (8), 3598–3604. doi: 10.1128/jvi.74.8.3598-3604.2000 10729135 PMC111869

[B20] Stokol T. Soboll H. G. (2019). Editorial: Current Research in Equid Herpesvirus Type-1 (EHV-1). Front. Vet. Sci. 6. doi: 10.3389/fvets.2019.00492 PMC6965053 31998768

[B21] Sucharita S. Zhang K. van Drunen L. D. H. S. (2021). VP8, the Major Tegument Protein of Bovine Herpesvirus-1, is Partially Packaged During Early Tegument Formation in a VP22-Dependent Manner. Viruses 13 (9), 1854. doi: 10.3390/v13091854 PMC8472402 34578435

[B22] Taddeo B. Roizman B. (2006). The Virion Host Shutoff Protein (UL41) of Herpes Simplex Virus 1 is an Endoribonuclease With a Substrate Specificity Similar to That of RNase A. J. Virol. 80 (18), 9341–9345. doi: 10.1128/JVI.01008-06 16940547 PMC1563938

[B23] Tang N. Zhang Y. Shen Z. Yao Y. Nair V. (2021). Application of CRISPR-Cas9 Editing for Virus Engineering and the Development of Recombinant Viral Vaccines. CRISPR J. 4 (4), 477–490. doi: 10.1089/crispr.2021.0017 34406035

[B24] Valyi-Nagy T. Shukla D. Engelhard H. H. Kavouras J. Scanlan P. (2007). ““Latency Strategies of Alphaherpesviruses: Herpes Simplex Virus and Varicella-Zoster Virus Latency in Neurons,” in Latency Strategies of Herpesviruses. Eds. Minarovits J. Gonczol E. Valyi-Nagy T. (Boston, MA: Springer US).

[B25] Van Cleemput J. Koyuncu O. O. Laval K. Engel E. A. Enquist L. W. (2021). CRISPR/Cas9-Constructed Pseudorabies Virus Mutants Reveal the Importance of UL13 in Alphaherpesvirus Escape From Genome Silencing. J. Virol. 95 (6), e02286–20. doi: 10.1128/JVI.02286-20 PMC8094956 33361431

[B26] Velusamy T. Gowripalan A. Tscharke D. C. (2020). CRISPR/Cas9-Based Genome Editing of HSV. Methods Mol. Biol. 2060, 169–183. doi: 10.1007/978-1-4939-9814-2_9 31617178

[B27] Weidner-Glunde M. Kruminis-Kaszkiel E. Savanagouder M. (2020). Herpesviral Latency-Common Themes. Pathogens 9 (2), 125. doi: 10.3390/pathogens9020125 PMC7167855 32075270

[B28] Wentink G. H. van Oirschot J. T. Verhoeff J. (1993). Risk of Infection With Bovine Herpes Virus 1 (BHV1): a Review. Vet. Q 15 (1), 30–33. doi: 10.1080/01652176.1993.9694365 8388593

[B29] Yang S. Wu Q. Wei Y. Gong C. (2019). CRISPR-Cas9 Delivery by Artificial Virus (Rrphc). Methods Mol. Biol. 1961, 81–91. doi: 10.1007/978-1-4939-9170-9_6 30912041

[B30] Zhang M. Fu S. Deng M. Xie Q. Xu H. Liu Z.. (2011). Attenuation of Bovine Herpesvirus Type 1 by Deletion of its Glycoprotein G and Tk Genes and Protection Against Virulent Viral Challenge. Vaccine 29 (48), 8943–8950. doi: 10.1016/j.vaccine.2011.09.050 21959327

[B31] Zhang F. Wen Y. Guo X. (2014). CRISPR/Cas9 for Genome Editing: Progress, Implications and Challenges. Hum. Mol. Genet. 23 (R1), R40–6. doi: 10.1093/hmg/ddu125 24651067

