# Supplementary figures and images for: Construction of BHV-1 UL41 Defective Virus Using the CRISPR/Cas9 System and Analysis of Viral Replication Properties
Source: Front Cell Infect Microbiol. 2022 Jul 8;12:942987. doi: 10.3389/fcimb.2022.942987 (PMC9304932; doi:10.3389/fcimb.2022.942987)

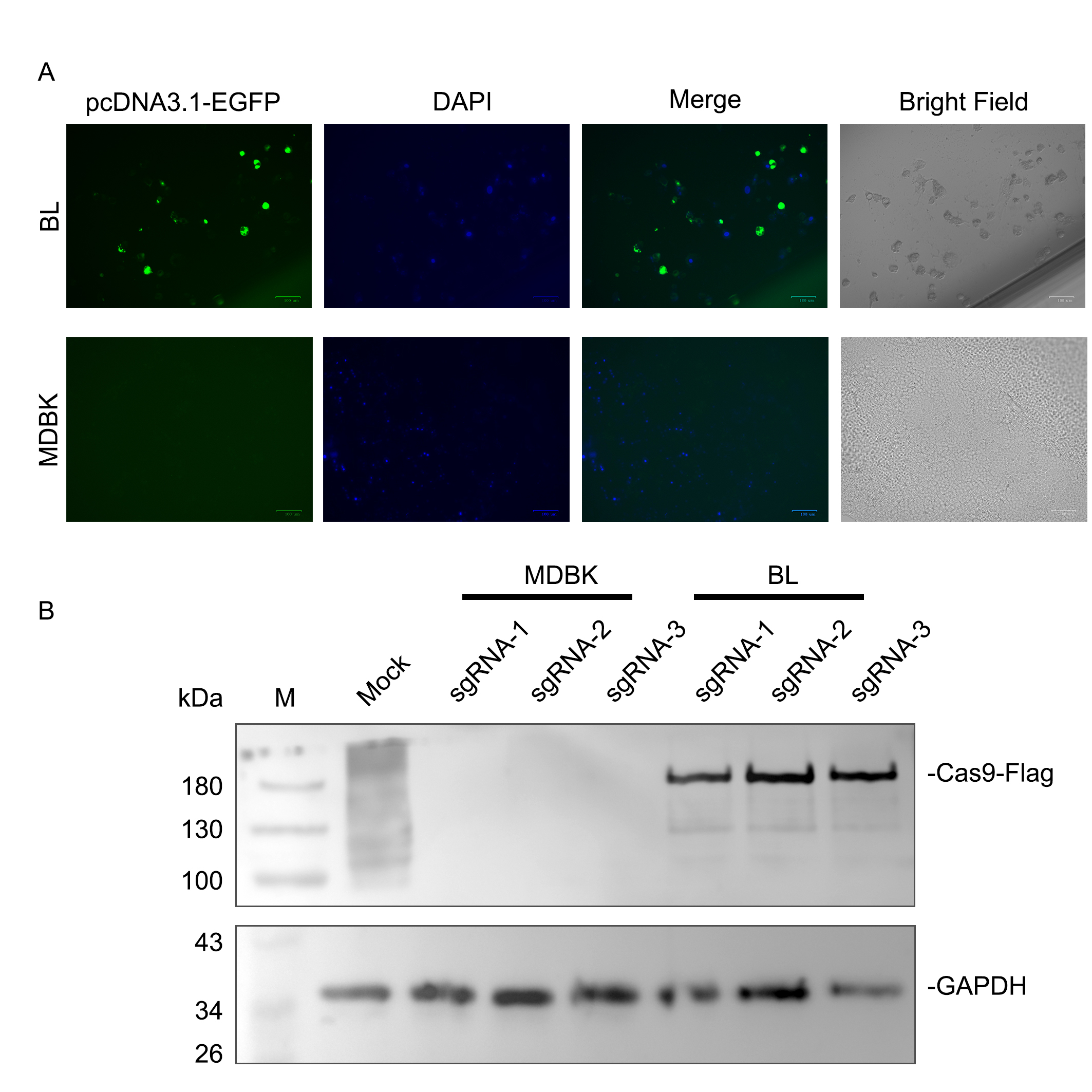

Supplement: Supplementary Figure 1 — The transfection efficiency of BL cells and MDBK cells. (A) Direct fluorescence analysis of the transfection efficiency of BL and MDBK. A plasmid of pcDNA3.1-EGFP transfected into BL and MDBK cells by 2× Max transfection reagent, (B) Western blot analysis of the transfection efficiency of BL and MDBK. Recombinant plasmids of pX330-sgRNAUL411-3 were transfected into BL and MDBK cells by Lipofectamine 3000 reagent (Invitrogen, USA). Cas9-Flag indicated the expression of recombinant plasmids, and GAPDH was the internal reference protein. [file Image_1.jpeg]
